# MicroRNA Variants miR-27a rs895819 and miR-423 rs6505162, but not miR-124-1 rs531564, are Linked to Endometriosis and its Severity

**DOI:** 10.3389/bjbs.2021.10207

**Published:** 2022-01-07

**Authors:** S. O. Jaafar, J. O. Jaffar, S. A. Ibrahim, K. K. Jarjees

**Affiliations:** ^1^ Department of Obstetrics and Gynecology, College of Medicine, Hawler Medical University, Erbil, Iraq; ^2^ Department of Obstetrics and Gynecology, Erbil Maternity Teaching Hospital, Erbil, Iraq; ^3^ Department of Food Technology, College of Agricultural Engineering Sciences, University of Salahaddin-Erbil, Erbil, Iraq

**Keywords:** endometriosis, microRNAs, polymorphism, variants, severity

## Abstract

**Background:** While different studies have investigated the association of SNPs with female reproductive disorders, a limited number of studies have investigated the effect of microRNAs variants in endometriosis. In this study, we evaluated the prevalence and the association of three different miRNAs variants including, miR-27a rs895819, miR-124-1 rs531564, and miR-423 rs6505162 with endometriosis to help further elucidate the importance of these variants in female reproductive disorders.

**Methods:** A total number of 440 women (220 cases and 220 controls) were included. DNA was extracted and genotyping of the SNPs was carried out by PCR.

**Results:** The results showed that rs895819 and rs6505162 had a significant association with endometriosis under the dominant, recessive, co-dominant, and allelic model, but rs531564 was not linked to endometriosis. Our results also imply a protective effect on endometriosis severity for AG genotype and G allele in rs895819 (*p* < 0.001), and also for AA and AC genotypes in rs6505162 with severity in endometriosis (*p* < 0.001). Moreover, Hardy–Weinberg equilibrium, haplotype frequency, and linkage disequilibrium between SNPs were performed.

**Conclusion:** miR-27a rs895819 and miR-423 rs6505162, but not miR-124-1 rs531564, are linked to endometriosis.

## Introduction

Infertility affects between 8 and 15% of couples of reproductive-age worldwide (1), the majority being residents of developing countries (2). Female infertility accounts for 37% of infertility cases (3). Researchers have shown a strong and consistent association between infertility and higher rates of endometriosis incidence among women with reproductive disorders and have considered endometriosis to be a leading cause of infertility (4). It is estimated that a quarter of reproductive-aged women suffer from endometriosis and accounts for up to 50% of female infertility cases (5). Endometriosis is defined as the presence of both ectopic endometrial stromal and glandular tissue and is thought to develop via reverse menstruation of viable endometrial tissue into the peritoneal cavity. Women with endometriosis mostly present with painful menstrual cramps, pain in the lower back and pelvis, pain when urinating during menstrual periods, and bleeding or spotting between menstrual periods. Intestinal pain and digestive problems have also been seen frequently among patients (6). While the mechanisms of endometriosis-related infertility remain unclear, factors including genetic, endocrine, and environmental factors have been considered to play an increasing role (7, 8).

MicroRNAs (miRNAs) are a class of small noncoding RNA molecules and are identified as key post-transcriptional regulators related to a myriad number of human diseases (9). Studies have shown dysregulation of hundreds of miRNAs in different disorders of the female reproductive system including endometriosis. miRNAs regulate a variety of normal and pathological cellular functions; hence, they might be potential candidates for therapeutic interventions in endometriosis (10).

Some single nucleotide polymorphisms (SNPs) in miRNA genes can impact their stability and affect the binding of miRNAs to their target genes, thus interfere with target gene recognition and expression (11). Many studies have investigated the association of SNPs with various female reproductive disorders (12–14), but a limited number of studies have investigated the effect of these SNPs in endometriosis. Therefore, in this study, we evaluated the prevalence and the association of three different miRNAs variants including, miR-27a rs895819A>G, miR-124-1 rs531564G>C, and miR-423 rs6505162A>C with endometriosis to help further understand the importance of these variants in reproductive disorders among females. To the best of our knowledge, this is the first study to investigate the association of these SNPs with endometriosis.

## Methods

A total of 440 women including 220 patients and 220 controls were recruited in this study. The patient group consisted of women who submitted to laparoscopic surgery for the evaluation of pelvic pain and/or infertility, and with histological confirmation of endometriosis and classification of the disease in stages I to IV according to the Revised American Society for Reproductive Medicine (15). The control group included endometriosis-free patients who underwent laparoscopic examination for other indications than infertility. Exclusion criteria for both patient and control groups were presence of systematic inflammation diseases and infections, ongoing pregnancy, history of pregnancy in the last 3 months, gynecological cancer, adenomyosis and other major systemic diseases. The study participants were enrolled from different private hospitals of Kurdistan Region-Iraq. The study has been approved by the high graduate committees of the College of Medicine, Hawler Medical University, Erbil, Kurdistan Region-Iraq. The informed consent documents were obtained from all subjects prior to sampling.

Blood samples were collected and stored at −20°C until use. Genomic DNA was extracted using Blood and Cell Culture DNA kit according to the manufacturer’s protocol. DNAs were analyzed by electrophoresis on 2% agarose gels. Three different SNPs were selected to be analyzed. The polymerase chain reaction-restriction fragment length polymorphism (PCR-RFLP) was performed to assess the polymorphism of miR-27a rs895819A>G, miR-124-1 rs531564G>C, and miR-423 rs6505162A>C. Table 1 shows general information of chosen SNPs and primers used in this study. The PCR conditions started with an initial denaturation at 94°C for 5 min, 35 cycles of denaturation of molecules at 94°C for 1 min, annealing at 60°C for rs895819, 67°C for rs531564, and 59°C for rs6505162 for 1 min, and the products were under extension at 72°C for 40 s, and then a final extension at 4°C for 5 min in Biorad Thermocycler. PCR products were separated on agarose gel and visualized by ethidium bromide staining.

**TABLE 1:** General information of selected SNPs obtained from dbSNP.

SNP	Position	Change	Primer sequences	Enzyme	Products length (bp)
miR-27a rs895819	chr5:160485411	NR_029501.1:n.40A>G	GAA​CTT​AGC​CAC​TGT​GAA​CAC​CAC​TTG​G TTG​CTT​CCT​GTC​ACA​AAT​CAC​ATT​G	DraIII	182, 155, 27
miR-124-1 rs531564	chr8:9903189	NR_024281.1:n.141C>G	TAC​AAT​TAA​GGC​ACG​CGG​TGA ACG​GAG​GAA​GGT​GTT​GAC​CC	Alw26I	410, 234, 176
miR-423 rs6505162	chr17:30117165	NR_029945.1:n.87A>C	CCC​CTC​AGT​CTT​GCT​TCG​TA AAGGGCGGGAATCAGGAC	RsaI	143, 124, 19

All statistical analyses were done using both SNPAlyze software (ver.8.1, Dynacom, Japan) and SPSS (ver.22). Allele and genotype frequencies of the SNPs among control and case groups were compared and checked using Pearson *χ*2 statistic. Moreover, deviations from Hardy–Weinberg equilibrium (HWE) were tested using a *χ*2 goodness-of-fit test. Analyses were also performed assuming recessive, codominant, and dominant models of inheritance and crude odds ratio (OR), their 95% CI ranges. Haplotype analyses were done using rs895819, rs6505162, and rs531564 variants for all study samples according to the maximum-likelihood method with an expectation–maximization algorithm.

In addition, pairwise LD coefficients of |D′| and *r* ^2^ were assessed using SNPAlyze software version 8.1 (DYNACOM, Japan). When alleles are in linkage disequilibrium, haplotypes do not occur at the expected frequencies. Linkage disequilibrium between two alleles is related to the time of the mutation events, genetic distance, and population history. It can be used to improve the power of genetic association studies.

|D′| and *r* ^2^ measurements used to estimate LD. Large values of *r* ^2^ indicate stronger association between alleles, and lower values of *r* ^2^ indicate weaker association between alleles. LD analyses were done based on Hardy-Weinberg equilibrium model. The significance level of the statistical tests was selected to be less than 0.05.

## Results

No statically significant difference was observed in the age (mean age ± SD: 35.8 ± 4.1 vs. 36.1 ± 3.65) and BMI (Kg/m^2^) (29.81 ± 4.85 vs. 28.64 ± 5.23) of endometriosis patients compared with controls (*p* = 0.31) (*p* = 0.52). Our results revealed that the frequencies of SNP alleles in the control group are in accordance with the Hardy–Weinberg equilibrium (Table 2).

**TABLE 2:** Allele and genotype distribution frequencies of SNPs in the case and control groups.

SNP	Model	Case	Control	X^2^	OR (95%CI)	*p*-value
rs895819	Allele A	320 (73)	252 (57)	22.38	0.50 (0.38–0.67)	***p* < 0.001**
	Allele G	120 (27)	188 (43)			
	Co-dominant (AG v AA+GG)	96 (44)	100 (45)	24.88	0.92 (0.63–1.35)	***p* < 0.001**
	Dominant (AA v AG+GG)	112 (51)	76 (35)	11.39	0.51 (0.35–0.76)	**0.001**
	Recessive (GG v AA+AG)	12 (5)	44 (20)	20.95	0.23 (0.11–0.45)	***p* < 0.001**
		† = 0.13	¥ = 0.29			
rs531564	Allele G	288 (65.5)	288 (65.5)	0.02	1.02 (0.77–1.34)	0.88
	Allele C	152 (34.5)	152 (34.5)			
	Co-dominant (GC v GG+CC)	106 (48)	92 (43)	2.01	1.29 (0.88–1.88)	0.36
	Dominant (GG v GC+CC)	91 (43)	98 (44.5)	0.59	1.16 (0.79–1.69)	0.44
	Recessive (CC v GG+GC)	23 (11)	30 (14)	0.78	0.76 (0.43–1.37)	0.37
		† = 0.33	¥ = 0.26			
rs6505162	Allele A	275 (62.5)	327 (74)	16.36	1.80 (1.35–2.40)	***p* < 0.001**
	Allele C	165 (37.5)	113 (26)			
	Co-dominant (AC v AA+CC)	115 (52)	89 (40)	17.16	1.35 (0.93–1.96)	***p* < 0.001**
	Dominant (AA v AC+CC)	80 (36)	119 (54)	15.45	2.14 (1.46–3.14)	***p* < 0.001**
	Recessive (CC v AA+AC)	25 (11)	12 (5)	6.33	2.42 (1.19–4.92)	**0.012**
		† = 0.08	¥ = 0.37			

^†^Indicates HWB *p*-value for cases. ^¥^Indicates HWB *p*-value for controls. Bold values are statistically significant values p<0.05.

Further analysis showed that rs895819 and rs6505162 had a significant association with increased rates of endometriosis in patients compared with the control group under the dominant, recessive, co-dominant, and allelic model, but miR-124-1 rs531564 was not linked to endometriosis. Allele and genotype distribution frequencies of every four SNPs in the case and control groups are shown in Table 2.

A subgroup analysis was conducted to evaluate the frequency and links between rs895819 and rs6505162 genotypes and patients with mild (*N* = 112) and severe endometriosis (*N* = 108) (Table 3). Significant differences were observed in AG and AA genotype prevalent among the subgroups regarding miR-27a rs895819 and polymorphism (*p* < 0.001). Our results also revealed a protective effect on endometriosis severity for AG genotype (65.1% vs. 21.2) and G allele (38 vs. 16.3%). The findings also showed a significant association of AA and AC genotypes in rs6505162 with severity in endometriosis (*p* < 0.001). Moreover, we found a significant protective effect for AC genotype (64.2% vs. 39.8 and C allele (46.5 vs. 28%).

**TABLE 3:** Allele and genotype distribution frequencies of SNPs in women with mild and severe endometriosis.

SNP	Model	Mild EN	Severe EN	*X* ^2^	OR (95%CI)	*p*-value
		*N* = 112	*N* = 108			
rs895819	Allele A	139 (62)	181 (83.7)	26.2	0.31 (0.20–0.49)	***p* < 0.001**
	Allele G	85 (38)	35 (16.3)			
	Co-dominant (AG v AA+GG)	73 (65.1)	23 (21.2)	44.8	0.14 (0.07–0.26)	***p* < 0.001**
	Dominant (AA v AG+GG)	33 (29.5)	79 (73.1)	41.9	0.15 (0.08–0.27)	***p* < 0.001**
	Recessive (GG v AA+AG)	6 (4.4)	6 (5.5)	0.04	1.03 (0.32–3.32)	0.94
rs6505162	Allele A	120 (53.5)	155 (71.7)	15.52	0.45 (0.30–0.67)	***p* < 0.001**
	Allele C	104 (46.5)	61 (28.3)			
	Co-dominant (AC v AA+CC)	72 (64.2)	43 (39.8)	22.0	0.36 (0.21–0.63)	***p* < 0.001**
	Dominant (AA v AC+CC)	24 (21.5)	56 (51.8)	21.99	0.25 (0.14–0.45)	***p* < 0.001**
	Recessive (CC v AA+AC)	16 (14.2)	9 (8.3)	1.93	0.54 (0.23–1.29)	0.16

EN, endometriosis.

To evaluate the association of these variants with endometriosis, haplotype analyses were done using, rs531564, rs6505162, and rs895819 variants for all study samples. The results showed a significant association between five haplotypes (GGA, GCA, CCA, CAG, and CCG) and endometriosis status. The GAA and corresponding to rs531564, rs6505162, and rs895819 was the most prevalent haplotypes among cases and controls, whereas the GCG haplotypes was the least prevalent haplotype among them. The frequencies of estimated haplotypes between cases and controls are shown in Table 4. Additionally, there was no LD between the rs895819, rs6505162, and rs531564 SNPs based on the measured D′ and *r* ^2^ parameters.

**TABLE 4:** Distribution of haplotype blocks in endometriosis patients and controls.

rs531564	rs6505162	rs895819	Overall frequency %	Case frequency %	Control frequency %	OR (95% CI)	*p*-value
G	A	A	28.8	29.3	0.16	1.06 (0.80–1.42)	0.661
G	A	G	18.7	21.7	0.16	0.65 (0.46–0.90)	**0.010**
G	C	A	12.5	7.8	0.16	1.92 (1.23–2.99)	**0.003**
C	A	A	11.7	12.2	0.16	0.75 (0.44–1.27)	0.285
C	C	A	11.7	7.0	0.16	2.02 (1.40–2.93)	***p* < 0.001**
C	A	G	8.5	11.0	0.16	0.54 (0.34–0.86)	**0.009**
G	C	G	5.3	6.7	0.16	0.74 (0.34–1.58)	0.442
C	C	G	2.5	3.3	0.16	0.17 (0.03–0.80)	**0.012**

## Discussion

We report significant associations of rs895819 and rs6505162 SNPs with higher rates of endometriosis, but no link with rs531564. The significance of miRNAs and their variants in different diseases have been indicated frequently (16, 17), whereas several studies have specifically investigated the effects of miRNAs variants in female reproductive disorders, a few studies have been focused to determine the role of miRNAs variants in endometriosis (18, 19).

Specific miRNAs have been identified as potential biomarkers for this disease in multiple studies. It has been reported that these miRNAs are linked to target genes and key pathways in pathophysiology of endometriosis. Cho et al. (20) in a study investigating the frequency of miRNA let-7a–f and miR-135a,b reported significantly decreased rates of circulating let-7b and miR-135a in women with endometriosis compared with controls and concluded that the combination of serum let-7b, 7d, and 7f levels could be served as a diagnostic marker for endometriosis. Similarly, Burney and coworkers showed that in endometriosis cases different miRNAs including miR-34c-5p, miR-9, miR-34b are down regulated in comparison to healthy cases (21). In another study, of 667 miRNAs that were studied in women with endometriosis, two miRNAs including miR-483-5p and miR-629 were highly downregulated in patients compared with controls and the authors of this study considered these miRNAs to have an important role in an early defect in the physiological activity of the proliferative endometrium, which eventually would lead to endometriosis (22). The importance of miRNAs including miR-145 (23, 24), miR-200b-3p (25), miR-92a (26), mir-185-5p (27), microRNA-520 (28), and miRNA-125b (29) has been indicated in various studies.

The important role of the rs895819, rs6505162, and rs531564 SNPs in female reproductive diseases have been also studied in several studies. Kin et al. showed that in women with implantation failures, miR-27aA>G had a significant association with a higher risk of recurrent pregnancy loss and IVF failure (30). Likewise, another case-control study of 99 women with at least two consecutive recurrent pregnancy losses (RPL) showed a strong association of rs895819 A/G with increased susceptibility to RPL (31). On the contrary, a recent study on a group of females with IVF failure reported that the frequency of miR-27aA>G has no difference between cases and controls (32). MiR-27a was also reported as a potential diagnostic marker for PCOS (33). 34. reported a role of miR‐27a‐3p and miR‐124‐3p in the pathology of endometriosis was demonstrated and their observation suggested that these miRNAs could represent non‐invasive markers of chronic endometritis. They concluded that these miRNAs could be exerted for therapeutic purposes in IVFF for assessment of endometrial quality.

Mir124 rs531564 gene polymorphism was found to be linked to a higher rate of cervical cancer (35), a finding supported elsewhere, which indicated that the G allele of miR-124 rs531564 polymorphism in cervical cancer patients was much less frequent than that in the controls, suggesting its possible role as a protective allele (36). The results from a meta-analysis study also showed the strong association of rs531564 with higher rates of cervical cancer under all genetic models (37). However, Danesh et al. (38), in a study consisted of 266 breast cancer patients and 288 control women, failed to link rs531564 polymorphism and the risk of breast cancer.

A strong association of mir423C>A with RPL was shown in a study investigating the role of this variant with unexpected RPL in China (39). The results from this study were further confirmed by Wang et al. who found that miR-423-CC/TT haplotype in pre-miR-423 may aggravate the risk of developing RPL by influencing the level of mature miR-423 and its target gene MESDC1 (40). In contrast, in another study in South Korea assessing the impact of four different miRNA variants including miR-27aA>G, miR-423C>A, miR-449bA>G, and miR-604A>G in a group of patients with idiopathic recurrent implantation failure (RIF) showed that miR-423C>A was not associated with the frequency of implantation failures among women (30).

This work represents an advance in biomedical science as it documents links between rs895819 and rs6505162 with endometriosis and it severity, and accordingly may be valuable tools in the diagnosis of this condition.

## Summary Tables

### What is Known About This Subject

• The importance of miRNAs and their target gene in endometriosis has been shown repetitively.
• There has been significant association between four different miRNAs variants including, miR-27a rs895819, miR-124-1 rs531564, and miR-423 rs6505162 and various female reproductive disorders.

### What This Paper Adds

• Our study showed a significant association of rs895819 and rs6505162 with increased rates of endometriosis among women.
• The rs895819 and rs6505162 variants indicated a significant association with severity of endometriosis under the dominant, co-dominant, and allelic model.
• The rs531564 didn’t indicate any association with endometriosis.

## Data Availability

The original contributions presented in the study are included in the article/supplementary material, further inquiries can be directed to the corresponding author.
